# Chitosan biopolymer promotes yield and stimulates accumulation of antioxidants in strawberry fruit

**DOI:** 10.1371/journal.pone.0203769

**Published:** 2018-09-07

**Authors:** Mosaddiqur Rahman, Julakha Akter Mukta, Abdullah As Sabir, Dipali Rani Gupta, Mohammed Mohi-Ud-Din, Mirza Hasanuzzaman, Md. Giashuddin Miah, Mahfuzur Rahman, Md Tofazzal Islam

**Affiliations:** 1 Department of Biotechnology, Bangabandhu Sheikh Mujibur Rahman Agricultural University, Gazipur, Bangladesh; 2 Department of Crop Botany, Bangabandhu Sheikh Mujibur Rahman Agricultural University, Gazipur, Bangladesh; 3 Department of Agronomy, Sher-e-Bangla Agricultural University, Sher-e-Banglanagar, Dhaka, Bangladesh; 4 Department of Agroforestry and Environment, Bangabandhu Sheikh Mujibur Rahman Agricultural University, Gazipur, Bangladesh; 5 Extension Service, West Virginia University, Morgantown, WV, United States of America; Huazhong Agriculture University, CHINA

## Abstract

Strawberry is a well-known source of natural antioxidants with excellent free radical scavenging capacity. This study determined the effects of chitosan application in field condition on plant growth, fruit yield and antioxidant activities in strawberry fruit. Foliar applications of chitosan on strawberry significantly increased plant growth and fruit yield (up to 42% higher) compared to untreated control. Increased fruit yield was attributed to higher plant growth, individual fruit weight and total fruit weight/plant due to the chitosan application. Surprisingly, the fruit from plants sprayed with chitosan also had significantly higher contents (up to 2.6-fold) of carotenoids, anthocyanins, flavonoids and phenolics compared to untreated control. Total antioxidant activities in fruit of chitosan treated plants were also significantly higher (ca. 2-fold) (p< 0.05) than untreated control. To the best of our knowledge, this is the first report of chitosan applied on field plants providing significant improvement of both yield and health benefiting biochemical contents in strawberry fruit. Further study on the elucidation of mechanisms involved with enhancement of growth, yield and biochemical contents by chitosan is needed to promote sustainable production of strawberry.

## Introduction

Consumption of strawberries, blueberries and brambles as fresh fruit containing bioactive compounds has gone up substantially during last decades due to their proven health benefits. Recent findings from several studies suggest that dietary sources of these bioactive compounds are highly beneficial to the health of humans [1, 2]. Strawberry (*Fragaria* × *annanasa*) is among the best sources of bioactive compounds containing anthocyanins, carotenoids, vitamins, flavonoids and phenolics with remarkable capacity of antioxidant activities [3–5]. Phenolic compounds such as anthocyanins, carotenoids and flavonoids present in strawberry have significant anticancer [2, 3, 6] and antioxidant [7] activities. Antioxidant capacity in strawberry fruit, however, directly or indirectly dependent on the level of flavonoid groups such as flavonols and anthocyanins [8]. Better health benefits to the consumers of strawberry can be obtained from elevated levels of these secondary metabolites. Excessive use of synthetic agrochemicals for enhancing fruit yield and contents of secondary metabolites led to serious environmental and health concerns especially in commodities like strawberries that are consumed fresh. This situation demands a novel eco-friendly and natural product based approach to improve yield and quality of strawberry fruit.

Chitosan, a biopolymer chemically derived from crustaceans and soluble in organic acids is one of a range of natural compounds that has shown efficacy against diseases in strawberries and other crops [9, 10]. It is considered environment-friendly for agricultural uses as it is easily degraded in the environment, and nontoxic to humans. Chitosan and its derivatives have been reported to elicit natural defense responses in plants, and have been used as a natural compound to control pre- and post-harvest pathogenic diseases [11]. Antimicrobial activities of chitosan against various phytopathogens have been reported [12]. Enhancement of storability and preservation of anthocyanin content in chitosan-coated strawberry fruit has been reported from multiple studies [10, 11]. Chitosan has been widely used as a coating agent of various fruit mainly for protection from post-harvest losses due to microbial infections [10, 11, 13, 14]. However, many investigators also reported that using chitosan as a foliar spray increased vegetative growth, yield and biochemical contents in plants [15–21]. Recent transcriptomic analysis reveals that chitosan induces expression of genes involved in multiple physiological processes including systemic acquired resistance, plant immune system, photosynthesis and hormone metabolism. It also influenced expression of heat-shock protein and re-programming of protein metabolism with an increment of storage proteins [22, 23]. However, information on field application of chitosan for enhancing yield and human health benefiting biochemical contents in small fruit is scant. Considering the potential of chitosan for enhancing plant growth, yield and quality, this study was conducted to investigate the performances of varying doses of chitosan on fruit yield and antioxidant contents in strawberry fruit in field condition. The specific objectives of the current study were to (i) evaluate the effects of four different doses of foliar spray of chitosan on growth and fruit yield of strawberry; (ii) asses the effects of foliar spray of chitosan on the induction of total carotenoids, anthocyanins, flavonoid and phenolic compounds in strawberry fruit; and (iii) determine the effect of chitosan spray on total antioxidant activities in strawberry fruit.

## Materials and methods

### Ethical statement

The lab and field experiments in this study were carried out following guidelines and recommendations of “Biosafety Guidelines of Bangladesh” published by Ministry of Environment and Forest, Government of the People’s Republic of Bangladesh (2005). However, the research works were strictly supervised by an advisory committee of research work of M.R. with the approval of Dean, Graduate studies, BSMRAU. The advisory committee monitored the research work considering the ethical issues.

### Experimental site

The study was carried out at the experimental field of Bangabandhu Sheikh Mujibur Rahman Agricultural University, Bangladesh (24.0379° N, 90.3996° E) during November 30, 2014 to March 25, 2015. Soil characteristics of the experimental field was shallow red brown terrace under Salna Series in Madhupur Tract (Agroecological zone 28) having a pH 6.7 [24]. The soil properties included 0.115% nitrogen, 21.35 ppm phosphorus, 0.24 meq. 100 g^−1^ soil exchangeable potassium and 1.70% organic matter. Tissue cultured seedlings of cv. Strawberry Festival were obtained from the Akafuji Agrotechnology, Rajshahi, Bangladesh. Agronomic and intercultural operations including fertilizer applications were performed as per commercial production guide.

### Experimental design and layout

The experiment was set in a randomized complete block design with three replications, and unit plot size was 75 cm × 150 cm. Plants were spaced 30.5 cm × 30.5 cm on the beds that were raised 30.5 cm above the main field with 86 cm aisles in between beds. Each plot had 8 plants in two adjacent staggered rows 30.5 cm apart. Thirty-day-old strawberry plug plants were transplanted on December 1, 2014 followed by providing intercultural operations and fertilization as needed. Data on plant growth parameters viz. number of leaves per plant, plant height (cm), leaf width (cm), leaf length (cm) and canopy diameter (cm) were recorded from every plant and averaged under a treatment at flowering stage (full blossom) [5]. Ripe fruit were harvested from individual plant under each treatment for consecutive 8 weeks, and the cumulative weight of all fruit from an individual plant was used as yield of fruit per plant for statistical analysis.

### Preparation and application of chitosan solution

Practical grade chitosan biopolymer (poly β-1,4-D-glucosamine) available in powder form was purchased from Sigma (Sigma-Aldrich, CAS Number 9012-76-4). It was commercially prepared by the alkaline deacetylation of chitin obtained from shrimp shells (*Pandalus borealis*). The degree of de-acetylation was ≥ 75% with low viscosity. Four different concentrations, 0, 125, 250, 500 and 1000 ppm of chitosan solution were prepared by measuring required amount of product followed by dissolving in 0.1 N HCl and diluting with distilled water with pH adjusted at 6.5 by 0.1 NaOH [25]. Freshly prepared chitosan solutions were applied onto strawberry plants in each experimental unit prior to flowering, and at 10% flowering stage by spraying up to run off in five different times with 10-d intervals starting from December 20, 2014 to February 22, 2015. Plants in the non-inoculated control plots were sprayed with equal volume of sterile water amended with equal volume of 0.1 N HCl and NaOH for adjusting pH at 6.5 (without chitosan).

## Assessment of chitosan effect on vegetative growth of strawberry plants

A total of six treatments with four different concentrations such as 0, 125, 250, 500 and 1000 ppm of chitosan and untreated control were assessed from three replicate plots. Each plot had 8 plants for a treatment. Vegetative growth of strawberry plant as affected by chitosan application was determined by measuring number of leaves per plant, plant height (cm), leaf width (cm), leaf length (cm) and canopy diameter (cm) with a ruler. Canopy diameter was assessed from the average of two perpendicular measurements through the center of each plant canopy included in a treatment. Runners produced by the transplants were removed as and when noticed on any plant. Fruit was harvested when the color of the fruit changed from pink to red. Fruit harvest was started on January 23, 2016 and continued until March 17, 2015. Immediately after harvest, sepals were removed, fruit were weighed and stored at −20°C for biochemical analyses from fruit flesh [26]. At the end of the fruit harvest, plants were carefully dug out of the ground with rhizosphere soil, root systems and shoots were separated, washed under running water, and fresh weight was recorded [5]. Root length (cm) was also recorded with a ruler. Dry weight of shoots and roots were taken after one week of drying at 60°C in an oven.

### Determination of antioxidants in strawberry fruit

In order to determine total carotenoids, flavonoids, anthocyanins, phenolics and total antioxidant activities a total of 16 randomly selected representative fruit samples from each treatment stored in refrigerator at −20°C in an airtight plastic container were defrosted and homogenized. One hundred grams of homogenized sample was used for biochemical analysis following the protocol described earlier [5]. Anthocyanins was extracted according the procedure described by Hughes and Smith (2007) [27] with some modifications. Briefly, one gram of homogenized fruit sample was used for extracting anthocyanins by using 5 mL 6M HCl:H_2_O:MeOH (7:23:70) and placed in the dark at 4°C for 24 h. Chlorophylls were separated from anthocyanins by adding 2 mL chloroform and 1 mL water to 2 mL fruit extracts. The reaction mixture was then centrifuged for 15 min at 5000 g. Total anthocyanin contents was determined spectrophotometrically by using 3 mL supernatant. Concentration of anthocyanins was estimated as cyanidin-3-*O*-glucoside equivalent from the absorbance at A530 (CT60, UV-visible spectrophotometer, PG Instruments) and a molar extinction coefficient of 30,000 l mol^-1^cm^-1^ [28]. Carotenoids content was estimated according the procedure described by Lachman et al. (2003) [29] with slight modifications. Briefly, acetone extract was obtained by mixing 5 mL of acetone with 2 g homogenized fruit sample in a glass vial and allowed to stand for 24 h at 4°C in the dark. The absorbance of the acetone extract was measured spectrophotometrically against acetone at 444 nm using 3 mL supernatant in a glass cuvette. Total carotenoids content was determined in mg g^-1^ of sample as lutein equivalent.

Aluminum chloride colorimetric assay method was used to determine total flavonoid in a spectrophotometer. Briefly, 1 mL methanol extract of strawberry fruit was added to 0.4 mL 5% sodium nitrate in a test tube whereas 1 mL methanol was taken instead of methanol extract of strawberry for blank reaction. After 5 minutes, 0.6 mL of 10% AlCl_3_.6H_2_O was added to the mixture. Two mL of 1M NaOH was added to the mixture at 6th minute followed by shaking thoroughly. The absorbance of the solution was then measured at 510 nm against the blank sample [30]. The absorbance data were compared to a standard curve of quercetin solutions to express total flavonoids content as (μg g^−1^ FW) quercetin. Folin-Ciocalteau method was used to determine total phenolic compounds in fruit spectrophotometrically. Briefly, 0.5 mL 10% (0.2 N) Folin–Ciocalteu reagent was added to each test tube containing 1 mL methanol extract of fruit sample whereas 1 mL methanol alone was used as blank. The test tubes were shaken for 10 s, covered and incubated for 15 min at room temperature (20 ± 2°C). Two and a half mL aqueous 700 mM sodium carbonate (Na_2_CO_3_) solution was added to each reaction mixture and vortexed. The reaction mixture was then covered and kept at room temperature for 2 h. The absorbance of the solution was measured at 765 nm against the blank sample. The absorbance data were compared to a standard curve prepared from gallic acid solutions and total phenolics were expressed as (μg g^−1^ FW) gallic acid equivalent.

### Antioxidant activity (DPPH radical scavenging assay)

The DPPH (1, 1-diphenyl-2-picryl hydrazyl, CalBiochem, Germany) radical scavenging assay was used to determine the antioxidant activity of strawberry fruit. This assay method is based on the reduction of DPPH that generates a stable free radical. This free radical has an odd electron, which gives a maximum absorption at 517 nm (purple color). One milliliter of DPPH solution (0.0788 g of 0.2 mM DPPH in 1L methanol) was added to 1mL methanol extracted supernatant of strawberry fruit sample in a test tube. The reaction mixture was then incubated at 25°C for 5 min followed by recording the absorbance at 517 nm [31]. The DPPH is reduced to DPPH-H by reacting with antioxidants from fruit extract, which results in the decrease of absorbance. results in decolorization Yellow color is developed due to the DPPH-H formation, which is proportional to the number of electrons captured. Solvents of DPPH solution without plant material served as the control. Methanol plant extracts (without DPPH) was used as blank. The following equation was used to calculate the DPPH radical scavenging activity of each sample (fruit extract).

$$\%\;Inhibition\;of\;DPPH\;radical\;activity=\frac{A\;Control-A\;Sample}{A\;Control}\times 100$$

### Data collection and statistical analyses

In order to determine the effect of chitosan on plant growth, leaf length (cm), leaf width (cm), plant height (cm), canopy diameter (cm), leaf number/plant, shoot fresh and dry weight (g), root fresh and dry weight (g), root length (cm), fruit weight (g/plant), individual fruit weight (g) and fruit yield was recorded [5]. Harvesting of fruit was started on January 23, 2016 and continued until March 17, 2018. For fruit quality assessment, anthocyanins mg (cyanidin-3-*O*-glucoside)/100g FW, carotenoids mg (lutein)/g FW, flavonoids μg (quercetin) /g FW, phenolics μg (gallic acid)/g FW, antioxidant μg (BHT) /g FW were measured from fruit of each experimental unit. The SPSS version 16 was used for analysis of variance of data collected from three replicates of each treatment. Each replication had 8 plants. Treatment means were separated using Fisher’s protected LSD test at (*p*≤0.05).

## Results

### Strawberry growth and yield enhancement by chitosan

#### Effect on plant canopy characteristic

Application of chitosan on the canopy of field grown strawberry plants significantly (*p*< 0.05) influenced leaf width (cm), leaf length (cm), leaf number / plant and canopy diameter compared to untreated control (Table 1). The highest leaf length (24.13 cm) was found in strawberry plants treated with 1000 ppm chitosan solution and the lowest (18.68 cm) was recorded in untreated control plants. Consistent with leaf length, leaf width of strawberry plants was also significantly increased in chitosan treated plants compared to untreated control. The highest leaf width (15.4 cm) was found in plants treated with 500 ppm chitosan solution and lowest (10.78 cm) in untreated plants. The leaf number and canopy diameter of strawberry plants in treated plots also showed significant variation from untreated control (Table 1). The highest leaf number (29.12)/plant was found in 1000 ppm chitosan solution treated plants and lowest (16.0) was in untreated control plants. Similar to leaf number, canopy diameter also had significant variation from the control treatments. The highest (36.5 cm) and lowest (29.12 cm) canopy diameter was recorded in 500 ppm chitosan treated plants and untreated control, respectively.

**Table 1:** Effect of chitosan on leaf length, leaf width, leaf number/plant and canopy diameter of strawberry cv. festival.

Treatment	Leaf length (cm)	Leaf width (cm)	Leaf number/plant	Canopy diameter (cm)
Control	18.68 ± 0.7^b^	10.78 ± 0.80^b^	16.0 ± 1.4^b^	29.12 ± 0.48^b^
Ch 125	22.85± 0.22^a^	13.7 ± 0.25^a^	23.8 ± 1.26^a^	33.8 ± 0.6^a^
Ch 250	23.82 ± 0.6^a^	13.5 ± 0.22^a^	24.33 ± 1.45^a^	33.9 ± 0.7^a^
Ch 500	23.13 ± 0.44^a^	15.4 ± 0.80^a^	27.83 ± 1.9^a^	36.5 ± 0.8a
Ch 1000	24.13 ± 1.0^a^	13.9 ± 0.06^a^	29.12 ± 1.94^a^	35.0 ± 1.0^a^

Values are means ± standard errors of three independent replications (n = 3). Different superscripted letters within the column indicate statistically significant differences among the treatments according to a Fisher’s protected LSD (least significance difference) test at p ≤ 0.05. Ch, chitosan solution in ppm concentration.

#### Effect on shoot and root growth

**Fig 1:**
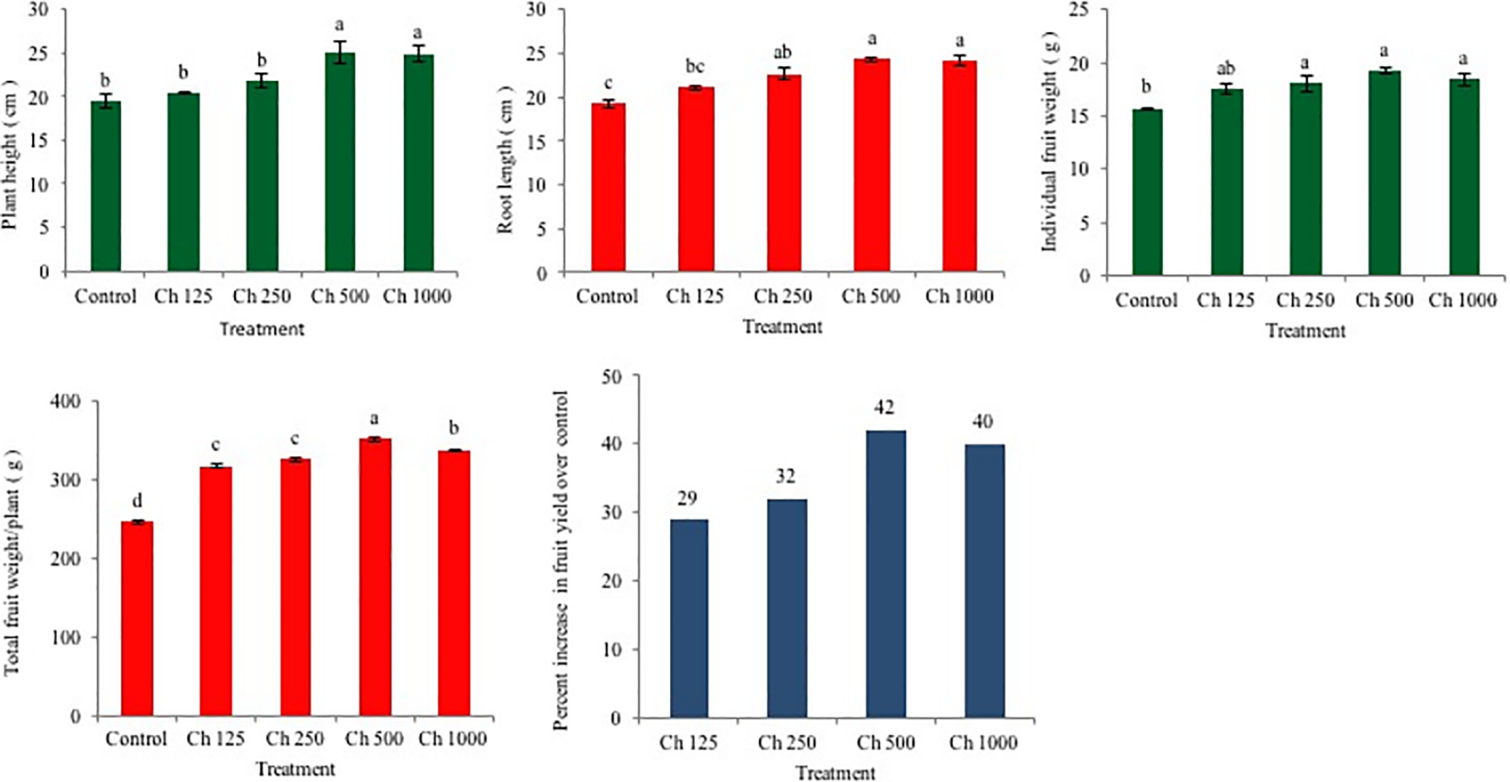
Average plant height (cm), root length (cm), individual (g), total fruit weight (g/plant) and increase in fruit yield of strawberry cv. Festival as influenced by the application of chitosan. Vertical bars on the top represent standard errors of the means (n = 24). Different letters indicate significant differences among the treatments at P≤ 0.05, according to a Fisher’s protected least significant difference test. Ch, chitosan solution in ppm concentration.

Root length and plant height increased significantly by the application of chitosan on canopy of field grown strawberry plants. The highest plant height (25.1cm) was obtained from 500 ppm chitosan solution treated plants and the lowest from untreated control (19.5 cm) plants (Fig 1). Similar to plant height, root length was also significantly (*p*< 0.05) influenced by different concentration of chitosan treatment (Fig 1). The largest gain in root length (24.33 cm) was recorded in 500 ppm chitosan solution treated plants whereas the lowest root length (19.25 cm) was in untreated control (Fig 1). There was no significant difference in plant height between 500 and 1000 ppm chitosan treatment indicating that 500 ppm may be the optimum concentration for achieving enhanced plant height through chitosan application. Similar trend was found for root length where optimum may lie in between 250 and 500 ppm.

#### Higher fresh and dry biomass production by chitosan treatment

Chitosan treatment positively and significantly influenced fresh and dry biomass production in strawberry plants (Table 2). The highest shoot fresh weight was found in 1000 ppm chitosan (235.5 g/plant) treated plants and the lowest shoot fresh weight was recorded in untreated control (134.5 g/plant) plants. Shoot fresh weight in 1000 ppm chitosan treatment was statistically superior to all other treatments. Shoot dry weight followed similar trend as fresh weight where 1000 ppm chitosan (65.6 g/plant) and untreated control (30.7 g/plant) plants had the highest and lowest shoot dry weight, respectively. Application of chitosan solution also significantly influenced root fresh and dry weight, which is consistent with shoot fresh and dry weight enhancement (Table 2). The highest root fresh weight was supported by 1000 ppm chitosan application on plant canopy (23.7 g/plant) compared with lowest root fresh weight in untreated control (12.6 g/plant). However, root fresh and dry weights were in disagreement with the highest root dry weight in 500 ppm chitosan treatment (10.5 g/plant) and the lowest in untreated control (7.2 g/plant) (Table 2).

**Table 2:** Effects of chitosan solution on fresh and dry biomass of strawberry cv. festival.

Treatment	Shoot fresh weight (g)	Shoot dry weight (g)	Root fresh weight (g)	Root dry weight (g)
Control	134.5 ± 0.7^e^	30.7 ± 0.8^d^	12.6 ± 0.3^c^	7.2 ± 0.3^c^
Ch 125	181.3 ± 2.9^c^	43.23 ± 1.4^c^	19.7 ± 0.8^b^	8.7 ± 0.4b^c^
Ch 250	168.0 ± 2.3^d^	47.33 ± 0.7^c^	17.33 ± 0.9^b^	10.2 ± 0.22^ab^
Ch 500	195.5 ± 2.0^b^	56.4 ± 1.22^b^	19.0 ± 0.6^b^	10.5 ± 0.43^a^
Ch 1000	235.5 ± 2.4^a^	65.6 ± 1.22^a^	23.7 ± 0.9^a^	9.9 ± 0.21^ab^

Values are means ± standard errors of three independent replications (n = 24). Different superscripted letters within the column indicate statistically significant differences among the treatments according to a Fisher’s protected LSD (least significance difference) test at p ≤ 0.05. Ch, chitosan solution in ppm concentration

#### Chitosan effect on fruit yield and yield component

**Fig 2:**
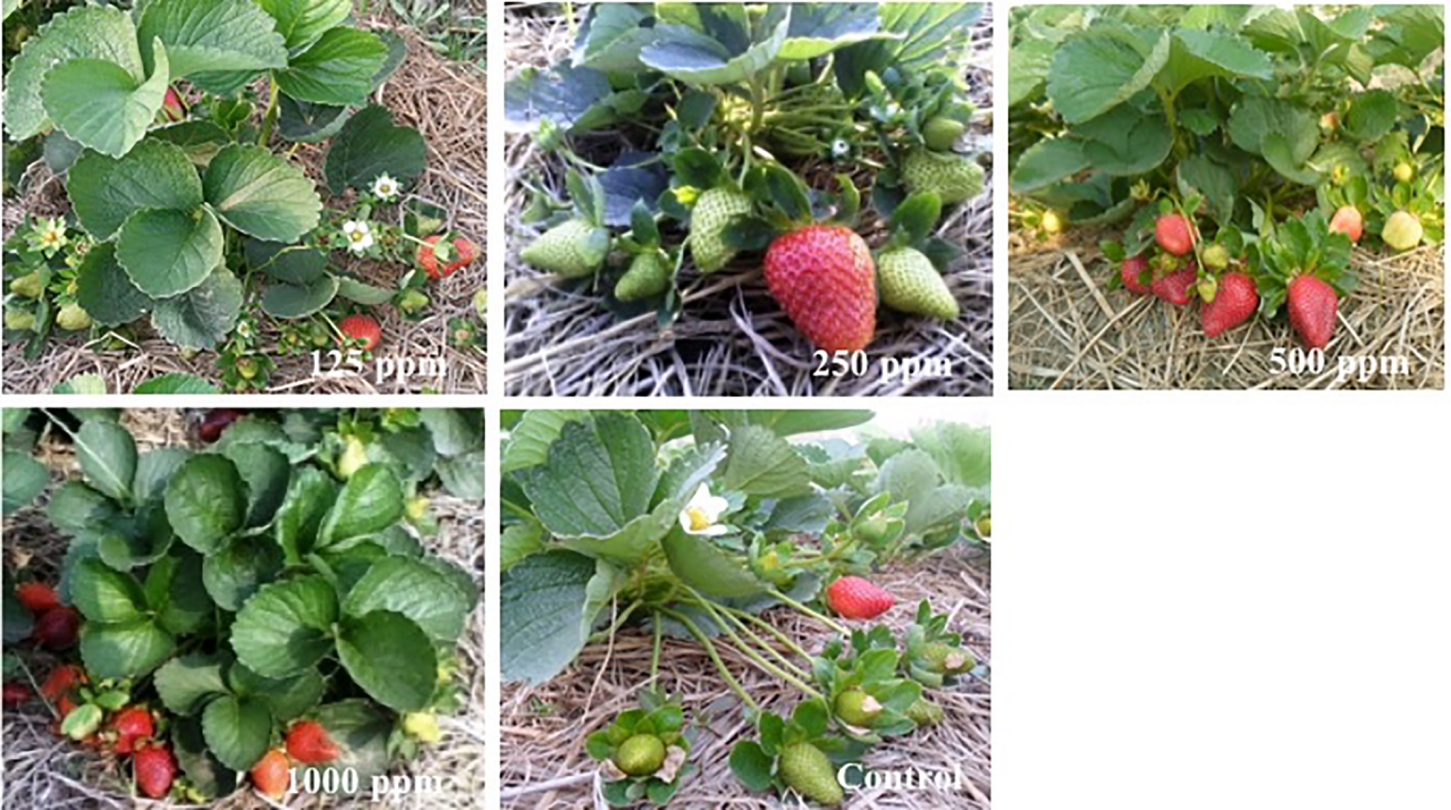
Effect of different doses of chitosan on vegetative and reproductive growth of strawberry cv. festival. All photographs were taken 54 days after transplanting of the plantlets.

Chitosan treatment significantly enhanced individual fruit weight and fruit yield of strawberry compared to untreated control. As chitosan application supported most of the growth parameters of strawberry plants, enhancement of fruit yield of strawberry was remarkable in most of the chitosan treatments. (Figs 1 and 2). However, both individual fruit weight (19.25 g/fruit) and total fruit weight per plant (351.25 g/plant) were the highest in 500 ppm chitosan treated plants compared to the lowest individual fruit weight and total fruit weight per plant being 15.7 g/fruit, 246.6 g/plant, respectively in untreated (Figs 1 and 2). Percent yield increase in 500 ppm chitosan treatment was calculated at 42% over untreated control (Fig 1).

### Effect of chitosan on the enhancement of total antioxidant activities and antioxidant contents in strawberry fruit

#### Total carotenoids and anthocyanins

**Fig 3:**
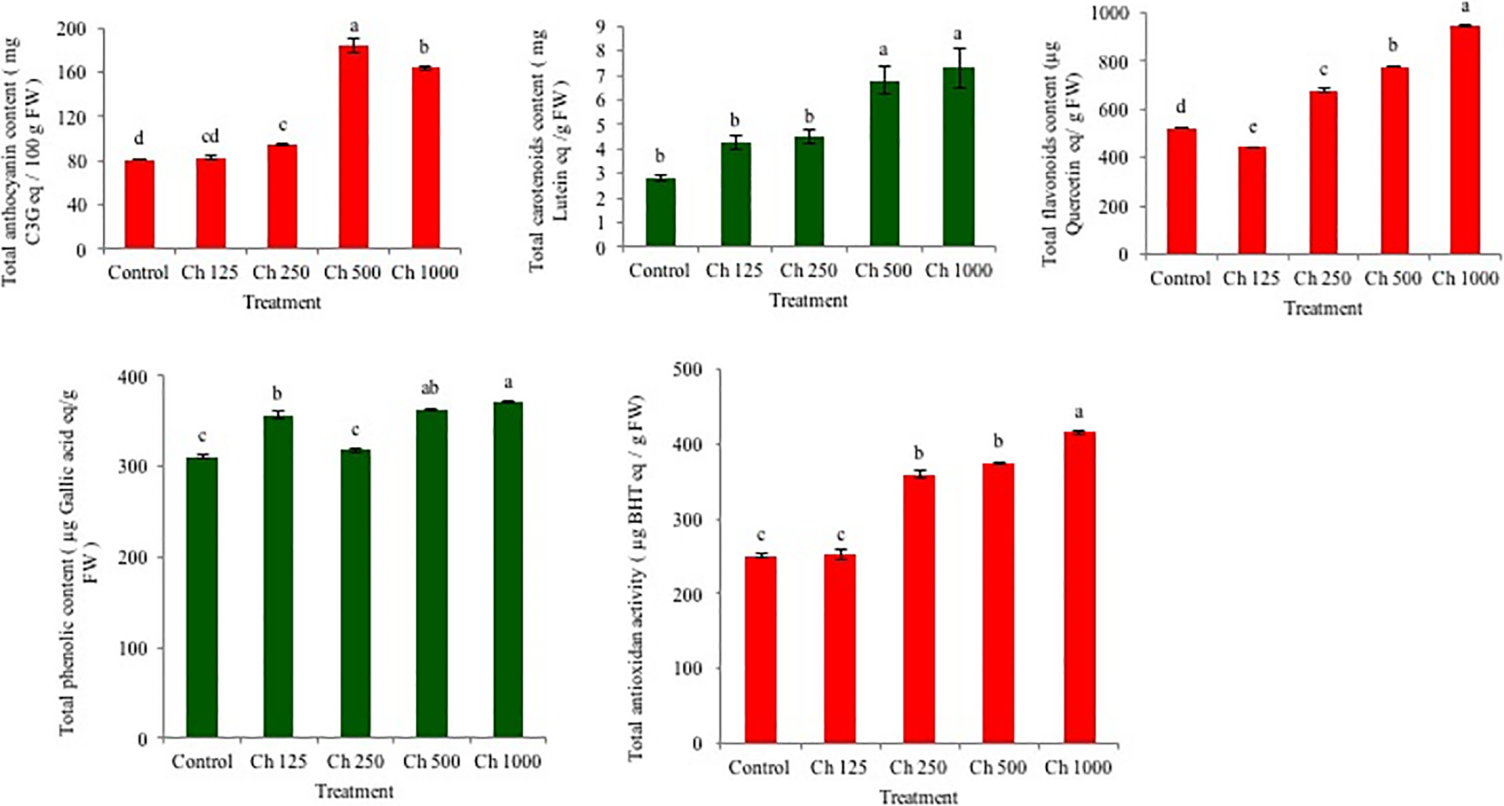
Enhancement of total anthocyanins (mg cyanidin-3-*O*-glucoside equivalent/100 g FW), carotenoids [mg (lutein) /g FW], total flavonoids (μg quercetin equivalent/g FW), phenolics (μg gallic acid equivalent/g FW) contents and antioxidant activity (μg BHT equivalent/g FW) of fresh strawberry cv. Festival by the treatment of chitosan. Bars represent standard errors of the means (n = 24). Different letters indicate significant differences among the treatments at P≤ 0.05, according to a Fisher’s protected least significant difference test. Ch, chitosan solution in ppm concentration.

Total carotenoids contents in strawberry fruit increased with spray application of chitosan in a dose-dependent manner up to 1000 ppm, which was significantly higher than those of the untreated plants. The highest carotenoids contents were estimated in fruit of 1000 ppm chitosan (7.73 mg lutein/g fruit) treated plants followed by 500 ppm chitosan (6.8 mg lutein/g fruit) that were 2.6- and 2.4-fold higher than that of untreated control (2.82 mg lutein/g fruit) (Fig 3). Chitosan spray application on the canopy of strawberry also significantly increased fruit anthocyanins contents in a dose-dependent manner but only up to 500 ppm compared to untreated control. The highest increase of anthocyanins (184.3 mg cyanidin-3-*O*-glucoside /100 g fruit) in strawberry fruit was supported by 500 ppm chitosan application on plant canopy that were statistically different from all other treatments, whereas the lowest anthocyanins contents were in untreated control plants (81.11 mg cyanidin-3-*O*-glucoside /100 g fruit) (Fig 3). Chitosan spray increased 2.3-fold higher anthocyanins contents in strawberry fruit than untreated control.

#### Total flavonoids and phenolics

Total flavonoids content of strawberry fruit was significantly and positively influenced by the application of different doses of chitosan compared to untreated control (Fig 3). Plants treated with 1000 ppm chitosan had the highest total flavonoids content (947.8 μg Quercetin/g fruit) in fruit, while the lowest total flavonoids content (523.7 μg Quercetin/g fruit) was found in untreated control. Similar to total flavonoids, total phenolics content in fruit was also enhanced by the application of different rates of chitosan compared to untreated control. The fruit produced by the plants treated with 1000 ppm chitosan solution had the highest total phenolics content (370.9 μg Gallic acid/g fruit), followed by 500 ppm chitosan (363.2 μg Gallic acid/g fruit) treatment (Fig 3). Fruit produced in untreated control plants had the lowest total phenolics content (310.4 μg Gallic acid/g fruit).

#### Total antioxidant activity

Total antioxidant activities of strawberry fruit obtained from both varied rates of chitosan treated and untreated control plants was assayed by utilizing DPPH method, and the results were expressed as butylated hydroxytoluene (BHT) equivalents per gram of strawberry fruit. The highest total antioxidant activity was quantified in strawberry fruit obtained from 1000 ppm chitosan (415.6μg BHT/g fruit) treated plants, which was significantly different from all other treatments, whereas the lowest antioxidant activity was in untreated control plants (250.9 μg BHT/g fruit) (Fig 3). These results reveal that application of chitosan on the canopy of strawberry could increase antioxidant activity in fruit up to 1.7-fold compared to untreated control.

## Discussion

Strawberry production and consumption has been showing an upward trend worldwide due to proven health benefits. In the US, per capita consumption of strawberries has increased from 2 pounds/person/year to approximately 8 pounds/person in recent years [32]. However, this high value fruit crop has also been on the list of dirty dozen due to high synthetic fertilizer and pesticide use for production and protection of the crop from multiple pests and diseases. Alternative approaches for growth promotion and pest management for strawberry is being explored for sustainable production worldwide as synthetic chemical inputs (fertilizer and pesticides) has created both environmental and health hazards. This is more relevant and significant for strawberry that is used for fresh consumption [33]. This study assessed the effect of an environment-friendly option for enhancing yield and functional properties of strawberry fruit through application of a biostimulant, chitosan. Results showed that application of low concentrations (ppm level) of a biostimulant, chitosan on the canopy of field grown strawberry plants significantly improved growth and yield with concurrent increase in various antioxidant contents and total antioxidant activities of strawberry fruit compared to untreated control. Quality and health benefits of strawberry fruit is considered intricately linked to the contents of total antioxidants and pigments such as anthocyanins. Enhancement of storability and preservation of anthocyanins content in chitosan-coated strawberry fruit has been reported by multiple investigators [10, 11,14]. To the best of our knowledge, this study for the first time demonstrated that foliar application of chitosan biopolymer can significantly increase both yield and contents of human health benefiting antioxidants in field grown strawberry fruit in a dose dependent manner. This is one of the few studies of its kind to determine the effects of chitosan application on field-grown strawberry plants influencing yield and contents of multiple antioxidants in fruit. Our findings suggest that both growth enhancement and accumulation of phenolic secondary metabolites in fruit are synergistically stimulated in strawberry by the foliar application of chitosan, which supports the alternative concept of coordination of growth and immunity pathways instead of mythical growth versus immunity tradeoff [34]. However, these results should be interpreted for other crops through field application of similar biostimulants in a representative agro-climatic condition for comparable cropping system.

One of the interesting findings of this study is that foliar application of chitosan at different doses improved growth of strawberry plants to some extent compared to untreated control. However, 500 ppm seems to provide highest growth stimulating effect compared to untreated control. The biostimulant chitosan is known to promote plant growth and development, and provide enhanced disease suppression capability to plants through multiple mechanisms including induced systemic resistance [10, 35]. Foliar application of varying doses of chitosan on strawberry canopy in this study stimulated all aspects of vegetative growth (leaf length, leaf number, shoot and root dry weights) with concurrent improvement of fruit yield and fruit quality compared with untreated control (Tables 1 and 2). Many investigators reported chitosan to control numerous pre- and post-harvest diseases, and increase yield of various ornamental as well as horticultural commodities in different parts of the world [35, 36]. Results from this study indicate that the rate of chitosan to promote growth and yield of field grown strawberries is milligram per liter concentration, which is in full agreement with previous findings [15, 18, 35]. Out of a few different concentrations tested in the current study, 500 ppm of chitosan solution provided the highest fruit yield (42% higher than untreated control) in ‘Strawberry Festival’ than that of untreated control (Fig 1), indicating this kind of treatment economically highly feasible and benign to the environment. As chitosan application could increase strawberry fruit yield up to 42% in this study, it should be of interest to assess how application of this natural stimulant benefit large scale commercial strawberry growers (Fig 1). Thus, an extensive field trial with multiple cultivars under different environmental conditions is needed before recommending large-scale field application. Although mechanism of growth and yield enhancement of strawberry by chitosan spray is not unraveled in this study, a recent RNAseq analysis reveals that chitosan spray induces overexpression of genes involved in photosynthesis, changes in programming of protein metabolism with an enhancement of various storage proteins and hormone metabolism [22].

Strawberry is particularly a good source of antioxidants with many fold higher free radical scavenging capacity compared to many other fruits such as grapefruit, grapes, oranges, kiwis, and mangoes [37]. Sojak, et al. (2015) [38] found that anthocyanins together with ascorbic acid were in part responsible for the antioxidant property of strawberry fruit. A wide variety of phenolics, including flavanols, proanthocyanidins, hydroxybenzoic and hydroxycinnamic acid derivatives, and hydrolysable tannins were also involved with strawberry antioxidant property. In our study, we found that application of low doses of chitosan on the canopy of strawberry plants produced 2.3 to 2.6-fold higher levels of anthocyanins and carotenoids in fresh strawberry fruit than that of untreated control (Fig 3). Similarly, chitosan application consistently produced remarkably higher levels of total flavonoids and phenolics in strawberry fruit (Fig 3), making this biostimulant an attractive agent for production of high quality and human health benefiting strawberry. Chitosan treatment of dark germinated barley sprouts (*Hordeum vulgare L*.) was also found to improve phenolic bioactive-linked anti-hyperglycemic function Similarly, exogenous application of chitosan on two species of basil (*Ocimum ciliattum* and *O*. *basilicum*) enhanced growth, yield and phenolic content and antioxidant activity [20]. Furthermore, Romanazzi et al. (2015) [39] reported that total polyphenols in several fruits had increased due to chitosan-coating that activated the key enzyme such as phenylalanine ammonia lyase (PAL) in the phenol synthesis pathway. Chitosan treatment of litchi fruit delayed changes in contents of anthocyanin, flavonoid, total phenolics by delaying the increase in PPO activity, and inhibited post-harvest decay partially [40]. However, to the best of our knowledge this study for the first time showed that low doses (mg/L) of chitosan spray on the canopy of field grown strawberry increased yield with concurrent significant improvement of the contents of multiple antioxidants and their activities in harvested fresh fruit.

**Fig 4:**
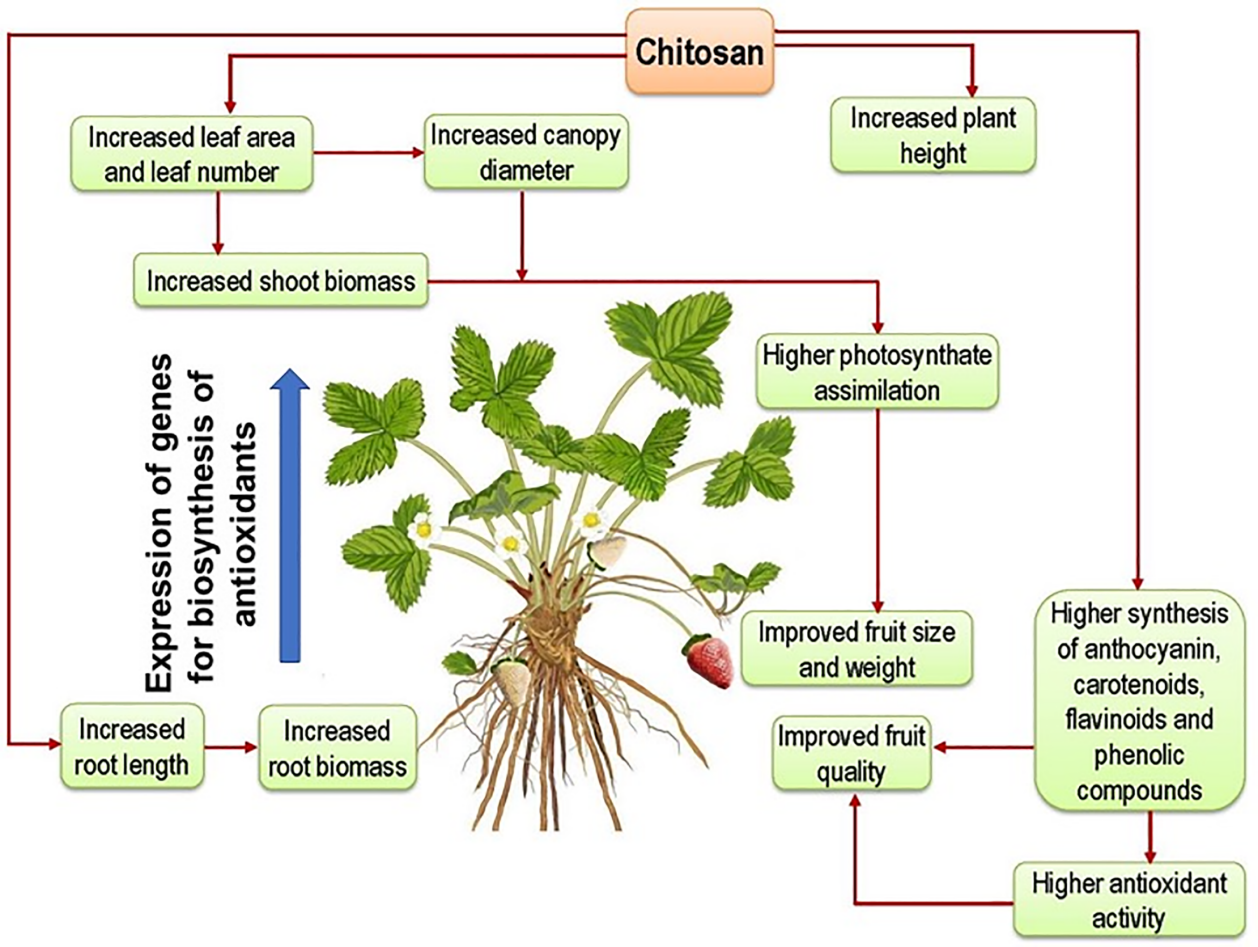
A hypothetical pathway of stimulation of fruit yield and accumulation of antioxidants in strawberry fruit cv. festival by the foliar application of chitosan.

Chitosan is a naturally occurring cationic biopolymer derived from chitin with unique bioactive properties in antimicrobial, plant growth induction and plant defense modulation. There are few studies with limited data on the positive effects of spray application of chitosan in reproductive stage of strawberry plants that remarkable increases (up to 2.5-fold) antioxidant contents in freshly harvested strawberry fruit (Fig 3). However, we recently reported the improvement of growth, fruit yield and content of antioxidants in strawberries by the treatment of two plant probiotic bacteria, *Paraburkholderia fungorum* BRRh-4 and *Bacillus amyloliquefaciens* BChi1 [5]. Previous study by Flores-Félix et al. (2015) [41] found that a member of the genus *Phyllobacterium* was an excellent plant probiotic with remarkable capacity of enhancing fruit yield and vitamin C contents in strawberry. Although mechanism of chitosan induced higher fruit yield and accumulation of antioxidants in strawberry fruit are not explored in the current study, we propose a hypothetical pathway in Fig 4. A further study is needed for validation of our proposed hypothesis [22, 23].

Utilization of biopolymer chitosan for quality enhancement of produce has multiple advantages such as safe for human health and environment, inexpensive, available in large quantities from deacetylation of naturally abundant chitin and biodegradable compared to synthetic inputs. In current study, we demonstrated that foliar application of chitosan significantly increased growth and fruit yield together with enhancement of human health benefiting properties of strawberry fruit by inducing higher production of total antioxidants, flavonoids, phenolics, carotenoids, and anthocyanins. Further study on the elucidation of mechanisms involved with growth, yield and biochemical contents enhancement in strawberry by chitosan would help better utilization of this biopolymer. Induction of gene expression in strawberries related to growth and secondary metabolites overproduction should be the major target of further study. Findings from the current study however, indicate that chitosan biostimulant obtained from naturally abundant chitin of crustaceans and fungal cell walls could be used as environment-friendly agent for sustainable production of high quality strawberry with no or little additional use of expensive synthetic inputs.
